# Newborn hearing screening coverage and detection rates of hearing impairment across China from 2008-2016

**DOI:** 10.1186/s12887-020-02257-9

**Published:** 2020-07-30

**Authors:** Xuelian Yuan, Kui Deng, Jun Zhu, Liangcheng Xiang, Yongna Yao, Qi Li, Xiaohong Li, Hanmin Liu

**Affiliations:** 1 National Office for Maternal and Child Health Surveillance of China, Department of Obstetrics, West China Second University Hospital, Sichuan University, Chengdu, Sichuan China; 2 National Center for Birth Defect Monitoring of China, West China Second University Hospital, Sichuan University, Chengdu, Sichuan China; 3 Department of Pediatrics, West China Second University Hospital, Sichuan University, Chengdu, Sichuan China

**Keywords:** Universal newborn hearing screening, Detection rate, Congenital hearing impairment, National

## Abstract

**Background:**

Newborn hearing screening (NHS) can reduce the economic and social burden of hearing impairment. To track the progress of the goal set by the National Program of Action for Child Development (NPACD) and to estimate the detection rates of hearing impairment, the present study examined NHS coverage in 2008–2010 and 2016 and the detection of hearing impairment across China in 2016.

**Methods:**

Licensed medical institutions across China were surveyed in 2012 and 2018 by the National Center for Birth Defects Monitoring of China to collect data for the 2008–2010 period and for 2016 on live births, initial screening rates (total and referral), secondary screening rates (total and referral), and rates of hearing impairment diagnosis among infants who were referred in the secondary screening. To calculate universal newborn hearing screening (UNHS) coverage, the number of newborns who received NHS within 4 weeks after birth was divided by the number of live births. The detection rate of hearing impairment was calculated by combining referral rates on primary and secondary screening with the rate of diagnosis.

**Results:**

National UNHS coverage increased from 29.9% in 2008 to 86.5% in 2016, with different regions showing different increases. During this period, the number of provinces with UNHS coverage over 90.0% increased from 2 to 17, with UNHS coverage in 2016 being substantially higher in eastern provinces (93.1%) than in western provinces (79.4%). In 2016, the detection rate of hearing impairment across the country was 0.23% (95% CI 0.15–0.25%), and it varied from 0.17% in western provinces to 0.22% in central provinces and 0.28% in eastern provinces. The lowest rate was 0.02% in Heilongjiang Province and the highest rate was 0.63% in Hainan Province.

**Conclusions:**

National UNHS coverage increased substantially from 2008 to 2016, although provinces and regions still showed differences. The detection rate of infant hearing impairment in China is comparable to that in other countries. A national individual-level information system is urgently needed in China to facilitate the integration of screening, diagnosis and treatment of infant hearing impairment, which may also lead to a more accurate estimate of the detection rate.

## Background

Hearing impairment in children is a serious obstacle to their development and education: it has been associated with delayed development of speech and language and cognitive skills, as well as with slow learning and difficulty in school [1]. According to the World Health Organization, impaired hearing affects 34 million children worldwide [2], including 0.5–5 of every 1000 newborns and infants [1]. In the US, hearing loss is associated with education costs of $115,600 per child [3].

Newborn hearing screening (NHS) can effectively enable the diagnosis of hearing impairment and intervention during the first 6 months, ensuring better outcomes for children [1, 4, 5]. Such screening may reduce special education costs by up to 37%, saving children from the need for up to 12 years of special classes for deaf children [6]. Starting with the work of Marion Downs in 1964 [7], the concept of universal newborn hearing screening (UNHS) has taken root in many developed countries [8–10], where UNHS coverage in 2016 was as high as 98.0% [9, 10]. NHS lags behind in many developing countries [11], reflecting a lack of financing for equipment; a lack of audiologists and newborn health workers, especially in rural areas; families’ concern over costs, which leads them to refuse NHS for their infants or refuse follow-up when indicated; and the discharge of neonates in less than 24 h, which prevents timely NHS [11].

In China, NHS was first adopted for high-risk neonates in Beijing in 1989, and UNHS was initiated in May 1996 in a few provinces [12]. In 1999, UNHS was incorporated into the lists of routine examinations of maternal and child health (MCH) care [12]. However, there were great disparities in the coverage of UNHS among the provinces, varying from 0 to 86% in 2004–2005, as published data show, mainly due to differences in economic development and people’s acceptance of hearing screening [12]. To promote the UNHS nationwide, a goal of UNHS ≥60% by 2020, which was one of the important contents of government performance appraisal, was set in the National Programme of Action for Child Development of China (NPACD) issued by the State Council in 2011 [13]. However, China has not yet established a regular reporting mechanism for hearing screening data yet, so the progress of national and subnational coverage of UNHS cannot be tracked and evaluated. In addition, the detection rate of hearing impairment in China remained unknown, resulting in the deficiency of evidence-based health resource allocation and planning.

With the aims of tracking the progress of the goal set by the NPACD and the estimation of detection rates of hearing impairment, the National Center for Birth Defects Monitoring of China (NCBDMC) conducted two surveys authorized by the National Health Commission of China (NHCC) in 2012 and 2018. Therefore, the baseline of the coverage of UNHS in 2008–2010 and its mid-term status were obtained, while the national and subnational detection rates of hearing impairment were estimated based on the survey in 2018.

## Methods

This study used a cross-sectional survey to collect the tabulated data on NHS and diagnosis, which were implemented in 2012 and 2018, respectively, by the medical institution. The data in the 2012 survey covered the period from 2008 to 2010, and the data in the 2018 survey covered the year 2016.

The two surveys were legal and mandatory data collections with the permission of the NHCC, and all the original data were tabulated data that did not involve individual information. The publication of the study has received permission from the NHCC. It meets the conditions of ethical exoneration according to the Council for International Organizations of Medical Sciences [14].

### Subjects

The two surveys included all licensed medical institutions providing NHS or a diagnosis of congenital hearing impairment in China. According to the *Regulation of Neonatal Disease Screening* promulgated by NHCC in 2009 [15], all the medical institutions providing NHS or hearing impairment diagnosis should be licensed by the provincial health administrative department. The licensed NHS institutions are responsible for the primary screening and secondary screening of newborns who were referred in the primary screening, and the referral of newborns who were referred in the secondary screening. However, licensed hearing impairment diagnosis institutions are responsible for those who were referred in the secondary screening. The process of hearing screening or diagnosis of hearing impairment should conform to the national technical guidelines for neonatal disease screening issued by the NHCC in 2010 [16]. The operation process specified by the technical guidelines can be seen in Additional file 1.

The 2012 survey included 3148 institutions that provided NHS and 95 that diagnosed infant hearing impairment, which were located in 30 provinces comprising 1657 districts/counties. The 2018 survey included 11661 institutions that provided NHS and 214 that diagnosed impairment, located in 31 provinces comprising 2664 districts/counties (Additional file 2).

### Data collection

This study collected the tabulated data from the two types of institutions using two unified questionnaires. The data on the number of live births, initial screening rates (total and referral), and secondary screening rates (total and referral) were collected from the licensed NHS institutions; data on rates of hearing impairment diagnosis among infants who were referred in the secondary screening were collected from the licensed hearing impairment diagnosis institutions.

**Fig. 1:**
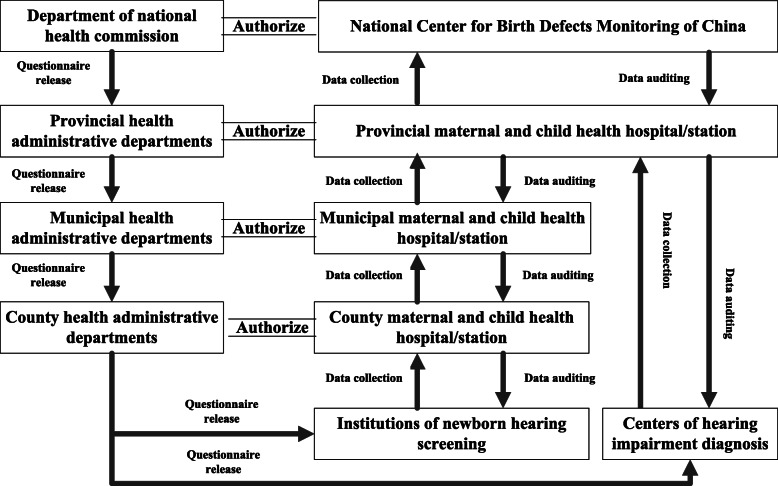
Flow chart of questionnaire release, collection and auditing

The questionnaires were released to provincial, municipal and county health administrative departments step by step, and ultimately to the licensed medical institutions. The staff in charge of the data management (usually the nurse) at each institution were responsible for completing the questionnaire. Then, the health administrative department at each level authorized the MCH hospital/station at the corresponding level to collect and audit the questionnaire (Fig. 1).

### Quality control

According to the requirements of the *Regulation of Neonatal Disease Screening* [15], all licensed institutions are responsible for routine data collection, although there is no need to report regularly. To ensure the accuracy of the data, hierarchical data auditing was implemented by a multilevel MCH hospital/station. This means that the county-level authorized MCH hospital/station should audit the data from the institutions in the jurisdiction, and municipal and provincial institutions and the NCBDMC audit the data step by step (Fig. 1). NCBDMC interviewed staff members cmpleting the questionnaires at 45 medical institutions in 3 provinces (Guangdong, Chongqing, and Shaanxi provinces) to further confirm the accuracy of the data.

### Statistical analysis

UNHS was defined as well-born babies who received NHS before discharge and babies in the neonatal intensive care unit who received hearing screening based on the results of an automatic auditory brainstem response test before discharge [17]. Diagnosis of hearing impairment was defined as well-born babies who were referred in the secondary NHS and were diagnosed with hearing impairment within 3 months after birth, and babies in the neonatal intensive care unit who are referred in the auditory brainstem response screening and were immediately diagnosed with hearing impairment.

To calculate UNHS coverage, the number of newborns who received NHS within 4 weeks after birth was divided by the number of live births. The detection rate of hearing impairment was calculated by combining the rates of primary and secondary screening referral with the rate of newborns who were referred in the secondary screening and were later diagnosed with hearing impairment (Additional file 3). This aggregate approach to calculating the detection rate was necessary because individual-level data on detection were unavailable. This aggregate detection rate does not take into account neonates admitted to the intensive care unit after birth.

Data were analysed for all 31 provinces in China, which were stratified into three geographic areas based on social and economic development [18]: eastern provinces included Beijing, Tianjin, Liaoning, Shanghai, Jiangsu, Zhejiang, Fujian, Shandong, and Guangdong; central provinces included Hebei, Shanxi, Jilin, Heilongjiang, Anhui, Jiangxi, Henan, Hubei, Hunan, and Hainan; and western provinces included Inner Mongolia, Guangxi, Chongqing, Sichuan, Guizhou, Yunnan, Shaanxi, Gansu, Qinghai, Ningxia, Xinjiang, and Tibet. All data were entered into Epidata 3.0 (The Epidata Association, Odense, Denmark) and analysed using R version 3.6.1 (R Foundation for Statistical Computing, http://www.r-project.org).

Estimated detection rates of hearing impairment in our study were calculated based on data from the 2018 survey. Rates were calculated for all provinces except Chongqing and Tibet, because data were unavailable on the number of Chongqing newborns diagnosed with hearing impairment among newborns who were referred in the secondary screening, and only 1979 of 50,896 live births received NHS in Tibet in 2016. National detection rates were estimated together with 95% confidence intervals (CIs), which were calculated by performing multiple sampling 1000 times based on provincial detection rates.

## Results

### UNHS coverage

**Fig. 2:**
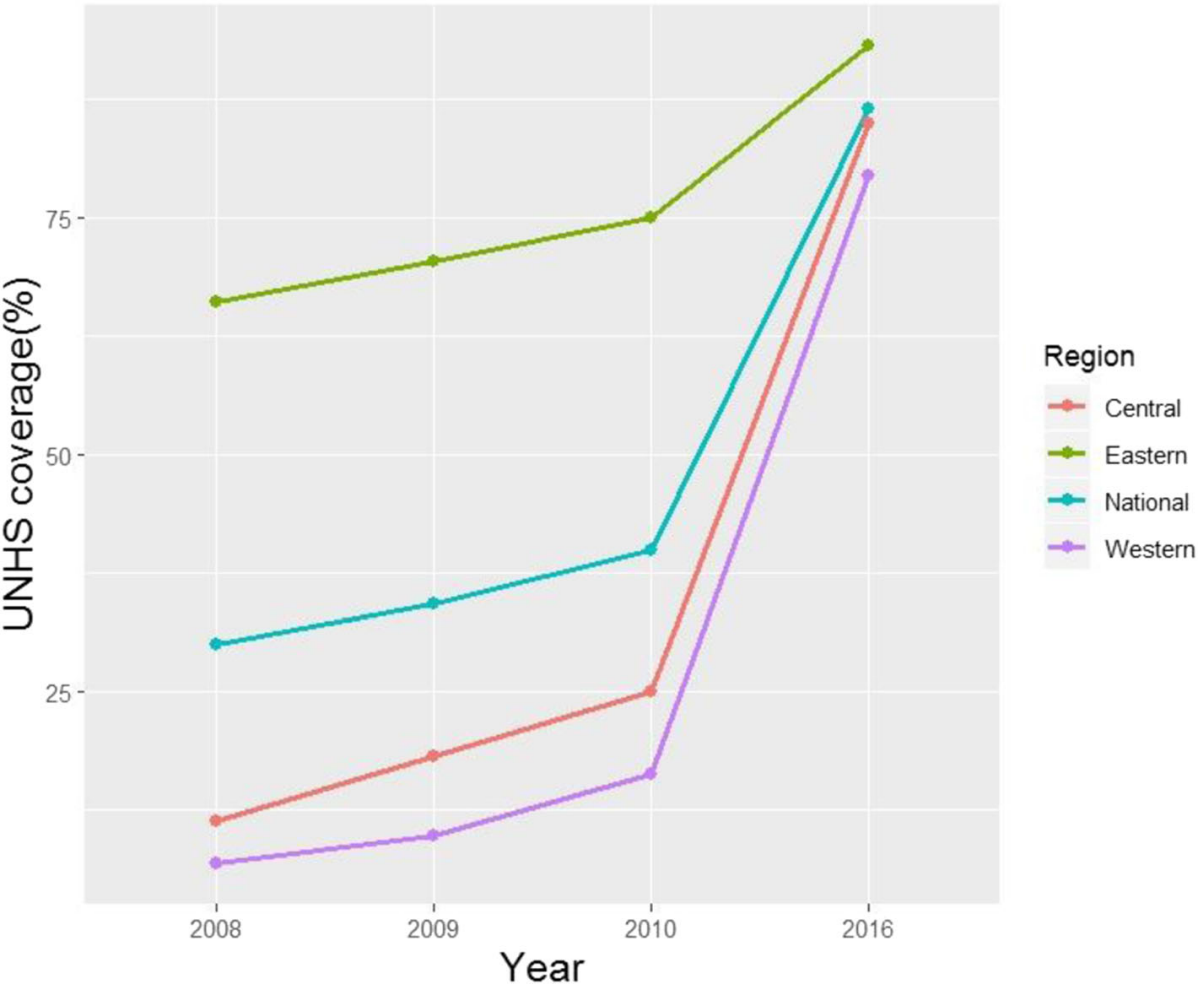
Universal newborn hearing screening (UNHS) coverage by region in China from 2008 to 2016. *Regions are defined in Methods

National UNHS coverage increased from 29.9% in 2008 to 86.5% in 2016, and substantial increases occurred in eastern provinces (66.0 to 93.1%), central provinces (11.2 to 84.9%), and western provinces (6.8 to 79.4%) (Fig. 2).

In 2008, 5 central provinces and 10 western provinces had UNHS coverage of less than 10%, and coverage in 13 of these provinces increased to at least 70% in 2016. In the same year, only two provinces, both in the eastern region, had UNHS coverage of more than 90%. In 2016, 17 provinces had UNHS coverage over 90.0%, comprising 8 eastern, 5 central, and 4 western provinces. Nevertheless, UNHS coverage remained lower for western provinces than for eastern provinces. In 2016, Guizhou had the lowest UNHS coverage of 46.1%, though that of Tibet was probably much lower (Table 1).

**Table 1:** Number of newborns receiving hearing screening (n) and the corresponding universal newborn hearing screening coverage by region and province of China in 2008 and 2016

Area	2008 ^a^		2016 ^b^	
	n	Coverage ^c^ (%)	n	Coverage ^c^ (%)
**National**	4,200,725	29.9	15,264,841	86.5
**Regional**				
Eastern	3,363,861	66.0	6,193,095	93.1
Central	584,198	11.2	5,301,210	84.9
Western	252,666	6.8	3,770,536	79.4
**Provincial**				
Beijing	151,835	88.5	244,731	95.9
Tianjin	69,926	64.4	132,654	99.4
Liaoning	211,699	65.1	333,811	94.4
Shanghai	174,435	100.0	207,659	98.2
Jiangsu	655,967	83.1	939,898	98.2
Zhejiang	515,557	85.0	711,994	97.0
Fujian	242,036	60.0	554,845	92.4
Shandong	908,824	94.6	1,570,953	97.3
Guangdong	433,582	27.9	1,496,550	83.5
Hebei	132,157	15.7	923,921	91.4
Shanxi	14,860	4.9	291,067	69.2
Jilin	12,029	5.7	183,103	94.1
Heilongjiang	57,676	22.8	197,917	95.5
Anhui	68,943	11.0	672,544	86.9
Jiangxi	28,822	5.2	494,026	80.0
Henan	57,606	4.9	1,106,339	80.0
Hubei	131,553	25.8	478,441	73.0
Hunan	79,336	12.2	830,235	98.2
Hainan	1216	1.1	123,617	91.5
Inner Mongolia	18,632	9.4	169,299	73.1
Guangxi	46,361	6.3	783,560	93.4
Chongqing	36,182	14.5	223,785	70.5
Sichuan	68,266	9.1	578,015	69.6
Guizhou	1790	0.5	227,496	46.1
Yunnan	8803	2.1	555,658	92.0
Shaanxi	35,801	13.1	458,343	89.1
Gansu	14,262	5.5	290,104	94.3
Qinghai	2122	2.7	42,144	68.3
Ningxia	4048	5.7	93,282	95.8
Xinjiang	16,399	5.6	346,871	86.5
Tibet	0	–	1979	3.9

^a^ There were 14,051,291 live births in 2008 in China, which was from the live births reported by midwives in each province

^b^ There were 17,644,192 live births in 2016 in China, which was from the live births reported by midwives in each province

^c^ Calculated as described in Methods

### Detection rate

**Fig. 3:**
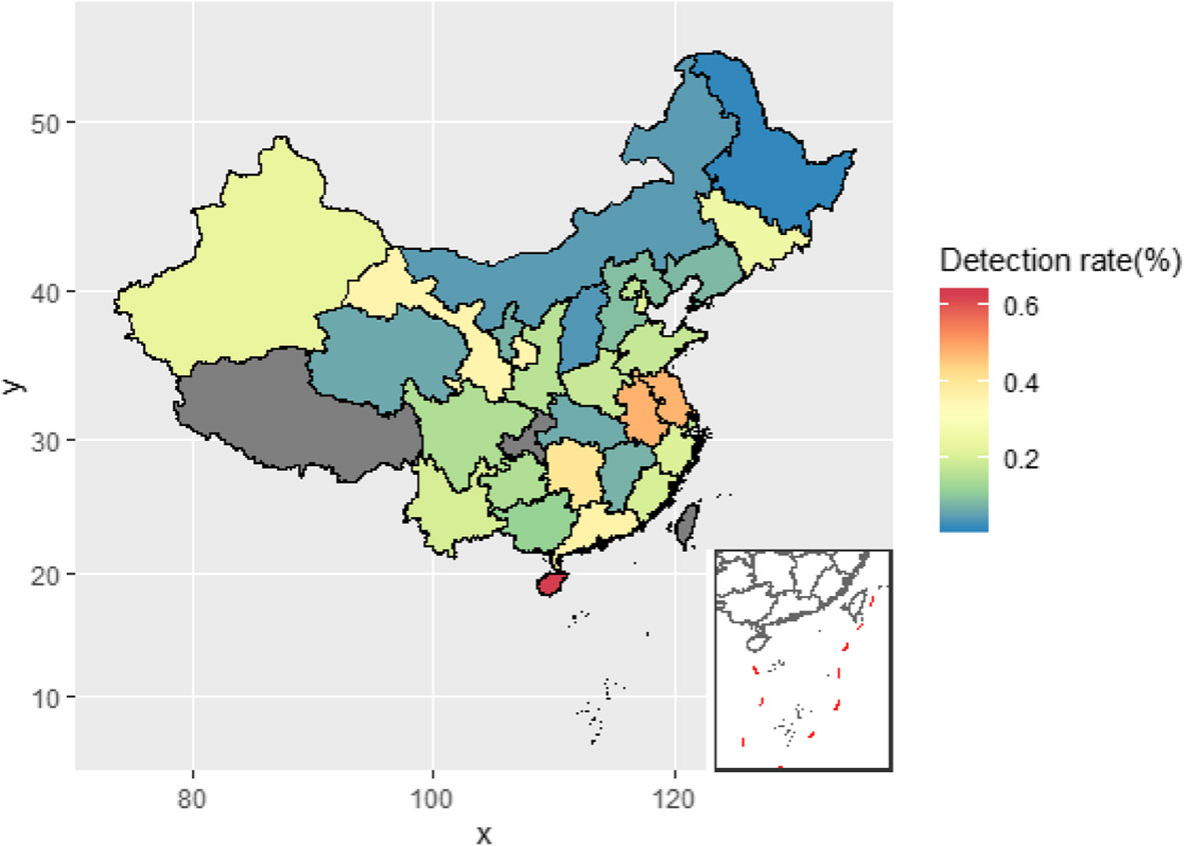
Detection rate of infant hearing impairment in 2016 in China, by province. *The map is created using R version 3.6.1 (R Foundation for Statistical Computing, http://www.r-project.org)

In 2016, a total of 28,167 cases were diagnosed as congenital hearing impairment by NHS, and the national detection rate of hearing impairment was 0.23% (95% CI 0.15–0.25%), which varied regionally from 0.17% (6186 cases) in the western provinces to 0.22% (10,272 cases) in the central provinces and 0.28% (11,709 cases) in the eastern provinces. In that year, the highest detection rate was 0.63% in Hainan Province, and the lowest was 0.02% in Heilongjiang Province (Fig. 3, Additional file 3).

## Discussion

Our results provide the most detailed insights to date into UNHS coverage and detection rates across China. They also provide provincial and national estimates of the detection rate of neonatal hearing impairment in the country. Our analysis indicates that from 2008 to 2016, the number of institutions providing UNHS and actual UNHS coverage improved substantially, especially in the central and western provinces. Nevertheless, different regions of the country continue to show disparities.

UNHS coverage in the study could benchmark China against other developing countries and potentially provide useful insights for UNHS improvement. UNHS coverage in some Chinese provinces reached nearly 100%, similar to the high coverage reported for the USA (98.0%) [9], UK (97.5%) [19], Poland (96.0%) [20] and parts of Italy where UNHS has been incorporated into law (99.3%) [21]. Such high coverage in developed countries has been attributed to programmes that subsidize the costs of UNHS or even make it free [19], the existence of national NHS guidelines [22] and Ministry of Health regulations, government funding for NHS to maternal and child health institutions, and productive collaboration among neonatologists, ear-nose-throat specialists, audiologists, nurses, midwives and parents [20]. Several of these factors may also help explain the substantial increase in UNHS coverage in China. In 2010, the NHCC issued a national plan for neonatal disease screening to promote UNHS [23], which aimed to reduce disparities in public health services [24]. In fact, Shaanxi Province [25] and some areas in certain provinces, such as Tianjin [26] and Beijing [27], began to provide free NHS in order to increase its acceptance by families. In addition, the NHCC launched a project in 2013 specifically aimed at neonatal disease screening in poverty-stricken areas, which stipulated improving the NHS network, training staff, subsidizing the NHS for families, and assessing the quality of the NHS [28]. The project was implemented in all central and western provinces, excluding Hainan Province, which meant that 21 provinces and 200 counties were covered. By 2014, the project had expanded to 364 counties [29]. The UNHS coverage of the 21 provinces increased from 21.5% in 2010 to 82.4% in 2016. In general, all of the above measures substantially contributed to the improvement of UNHS in China and the achievement of the NPACD goal (UNHS coverage at 60% in 2020) ahead of time.

At the same time, several Chinese provinces, such as Tibet and Guizhou, show quite low UNHS coverage, akin to that of several developing countries [11]. This low coverage has been attributed in some countries to lack of funding and equipment in medical institutions, a lack of medical personnel, and a generally low prioritization of NHS [11, 30]. Data indicate that 8 medical institutions provide NHS in Tibet and 155 in Guizhou; these numbers correspond to rates of 1.6 and 3.1 institutions per 10,000 live births, respectively, which is far below the national average of 6.6 per 10,000 across China as a whole, according to our unpublished report. Unpublished reports of the National Health Commission suggest that Guizhou has undertaken several measures to promote NHS including the acquisition and dissemination of NHS equipment and free training for hearing screening professionals. These measures may help explain why UNHS coverage in Guizhou increased from 0.5% in 2008 to 71.4% in 2018.

We report detection rates of neonatal hearing impairment at the provincial and national levels in China. Since UNHS coverage has reached 86.5%, our detection rates may be reasonable estimates of the actual incidence of newborn hearing impairment. We measured a national detection rate of 0.23% (95% CI 0.15–0.26%) in 2016. According to the data we acquired from the survey conducted in 2018, only 72.7% of neonates who were referred in the primary screening underwent secondary screening (secondary screening rate), and only 53.9% of neonates who were referred in the secondary screening were referred to medical institutions for diagnosis (referral rate). Given the lack of a national, individual-level information system, we had to collect all our data from licensed medical institutions providing diagnosis, which means that we missed patients who were diagnosed at unlicensed places. Therefore, our estimated referral rate may underestimate the true rate. Nevertheless, our estimate falls within the global incidence of 0.05–0.5% among neonates and infants [1]. This finding suggests that the data analysed in the present study can provide a reasonable basis for analysing neonatal hearing impairment in China, and it suggests that the country has succeeded in improving UNHS coverage and detection. On the other hand, the observation that rates of secondary screening and of diagnosis/treatment are substantially below 100% indicates lagging integration of diagnosis and treatment. They also reflect the urgent need for a national individual-level information system to monitor and address this integration gap. Province-level studies have confirmed that unified individual-level information systems can ensure the availability of timely, accurate and comprehensive data [31–33].

Several limitations of the study should be noted. First, although several methods of data quality control had been carried out, the UNHS coverage was estimated based on the tabulated data rather than individual-level data, which may affect the accuracy of the data. Therefore, we compared some provincial screening rates with those of previous studies, such as the rates in Guangdong [34], Hubei [35], and Hunan [36] provinces. Second, some inevitable factors could affect the accuracy of the detection rate. One is the low UNHS coverage of the year 2016 in some western provinces (such as Tibet and Guizhou), which could affect the representativeness of our estimated detection rate for the actual provincial detection rate. Then, rates of secondary screening and referral less than 100% may cause system error between estimated and actual detection rates. Cases attended secondary screening or referred to the licensed institutions, and others who did not may have different risks of hearing impairment, leading to different diagnostic rates, and affecting the accuracy of the estimated detection rate. In addition, although all the licensed medical institutions are required to be equipped and to perform hearing screening and diagnosis, in compliance with the national technical guidelines, the reported data could be affected by various methods of screening and different levels of operator experience.

## Conclusion

National UNHS coverage in China increased substantially from 2008 to 2016, which substantially contributed to the achievement of goals set by the NPACD, but geographic disparities remain. The detection rate of infant hearing impairment in China appears to be similar to that in many other countries. A national individual-level information system is needed to help integrate screening, diagnosis and treatment in China, which may also help determine a more accurate detection rate.

## Supplementary information

**Additional file 1.** The process of newborn hearing screening specified in national technical guidelines for screening of neonatal diseases.

**Additional file 2.** Coverage area of medical institutions that participated in the two surveys. *NHS, newborn hearing screening.

**Additional file 3.** Detection rate of newborn hearing impairment in 2016 in China, by province and region. *The data of cases diagnosed as hearing impairment in the table could not totally represent the actual data of cases diagnosed in 2016, as the data of cases diagnosed as hearing impairment in our study were collected from licensed medical institutions providing diagnostic/treatment. ^a^ Referral rate of primary screening was estimated from the formula: number of referral in primary screening / NHS number *100. ^b^ Referral rate of secondary screening was estimated from the formula: number of referral in secondary screening / number of secondary screens *100. ^c^ Diagnostic rate of hearing impairment was estimated from the formula: number of referral in secondary screening / number of cases diagnosed with hearing impairment * 100. ^d^ Detection rate of hearing impairment was estimated from the formula: (referral rate of primary screening / 100) * (referral rate of re-screening / 100) * (Diagnostic rate of hearing impairment / 100) *100. ^e^ The 95% confidence interval for this detection rate was 0.15 to 0.26%, which was estimated as described in Methods. ^f^ Chongqing and Tibet were not included in the analysis.

## Data Availability

The primary data set collected from medical institutions and analysed during the current study is available from the corresponding author. In addition, administrative consent must be obtained to share the data. Further information can be requested by e-mailing the principal investigator (lixiaohong82@scu.edu.cn ).

## Abbreviations

NHS: Newborn hearing screening
UNHS: Universal Newborn hearing screening
NHCC: National Health Commission of China
NPACD: National Programme of Action for Child Development
NCBDMC: National Center for Birth Defects Monitoring of China
OAE: Otoacoustic emission
AABR: Automatic auditory brainstem response
ABR: Auditory brainstem response
