# Supplementary material for: Newborn hearing screening coverage and detection rates of hearing impairment across China from 2008-2016
Source: BMC Pediatr. 2020 Jul 30;20:360. doi: 10.1186/s12887-020-02257-9 (PMC7391493; doi:10.1186/s12887-020-02257-9)
Supplement: Supplementary file 2 — Additional file 2. Coverage area of medical institutions that participated in the two surveys. *NHS, newborn hearing screening. [file 12887_2020_2257_MOESM2_ESM.docx]

| Data | 2008 | |  | 2016 | |
| --- | --- | --- | --- | --- | --- |
|  | Total | Covered  N (%) |  | Total | Covered  N (%) |
| Medical institutions providing NHS* | - | 3148(100.0) |  | - | 11661(100.0) |
| Medical institutions providing diagnosis/ treatment | - | 95 (100.0) |  | - | 214(100.0) |
| Provinces | 31 | 30 (96.8) |  | 31 | 31 (100.0) |
| Cities | 333 | 282 (84.7) |  | 334 | 328 (98.2) |
| Counties | 2859 | 1657 (58.0) |  | 2928 | 2664 (91.0) |
