# Supplementary material for: Newborn hearing screening coverage and detection rates of hearing impairment across China from 2008-2016
Source: BMC Pediatr. 2020 Jul 30;20:360. doi: 10.1186/s12887-020-02257-9 (PMC7391493; doi:10.1186/s12887-020-02257-9)
Supplement: Supplementary file 3 — Additional file 3. Detection rate of newborn hearing impairment in 2016 in China, by province and region. *The data of cases diagnosed as hearing impairment in the table could not totally represent the actual data of cases diagnosed in 2016, as the data of cases diagnosed as hearing impairment in our study were collected from licensed medical institutions providing diagnostic/treatment. a Referral rate of primary screening was estimated from the formula: number of referral in primary screening / NHS number *100. b Referral rate of secondary screening was estimated from the formula: number of referral in secondary screening / number of secondary screens *100. c Diagnostic rate of hearing impairment was estimated from the formula: number of referral in secondary screening / number of cases diagnosed with hearing impairment * 100. d Detection rate of hearing impairment was estimated from the formula: (referral rate of primary screening / 100) * (referral rate of re-screening / 100) * (Diagnostic rate of hearing impairment / 100) *100. e The 95% confidence interval for this detection rate was 0.15 to 0.26%, which was estimated as described in Methods. f Chongqing and Tibet were not included in the analysis. [file 12887_2020_2257_MOESM3_ESM.docx]

| Area | Referral rate | | | | | | | | | | Diagnostic of hearing impairment ^c^ | | | Detection rate of hearing impairment ^d^ | |
| --- | --- | --- | --- | --- | --- | --- | --- | --- | --- | --- | --- | --- | --- | --- | --- |
|  | Primary screening ^a^ | | | | Secondary screening ^b^ | | | | | |  |  |  |  |  |
|  | n | (%) | |  | | | n | | (%) | | n* | | (%) | | (%) |
| **National** | 1514348 | 9.9 | |  | | | 160470 | | 14.6 | | 28167 | | 15.9 | | 0.23 ^e^ |
| **Regional** |  |  | |  | | |  | |  | |  | |  | |  |
| East | 666480 | 10.8 | |  | | | 80527 | | 17.9 | | 11709 | | 14.7 | | 0.28 |
| Central | 492590 | 9.3 | |  | | | 44860 | | 11.9 | | 10272 | | 19.9 | | 0.22 |
| West | 355278 | 9.4 | |  | | | 35083 | | 12.9 | | 6186 | | 13.7 | | 0.17 |
| **Provincial** |  |  | |  | | |  | |  | |  | |  | |  |
| Beijing | 19355 | 7.9 | |  | | | 2475 | | 16.1 | | 516 | | 13.4 | | 0.17 |
| Tianjin | 13956 | 10.5 | |  | | | 1040 | | 11.0 | | 200 | | 19.2 | | 0.22 |
| Liaoning | 30116 | 9.0 | |  | | | 2746 | | 15.3 | | 420 | | 5.9 | | 0.08 |
| Shanghai | 17876 | 8.6 | |  | | | 2153 | | 13.7 | | 805 | | 14.1 | | 0.17 |
| Jiangsu | 82441 | 8.8 | |  | | | 13351 | | 19.6 | | 2955 | | 27.8 | | 0.48 |
| Zhejiang | 55593 | 7.8 | |  | | | 7270 | | 14.4 | | 1384 | | 18.3 | | 0.21 |
| Fujian | 52664 | 9.5 | |  | | | 9636 | | 22.1 | | 896 | | 9.6 | | 0.20 |
| Shandong | 124086 | 7.9 | |  | | | 13332 | | 12.1 | | 1467 | | 18.5 | | 0.18 |
| Guangdong | 270393 | 18.1 | |  | | | 28524 | | 23.9 | | 3066 | | 8.5 | | 0.37 |
| Hebei | 94087 | 10.2 | |  | | | 6885 | | 10.4 | | 1434 | | 8.5 | | 0.09 |
| Shanxi | 34940 | 12.0 | |  | | | 1934 | | 10.0 | | 169 | | 3.1 | | 0.04 |
| Jilin | 20350 | 11.1 | |  | | | 1917 | | 14.3 | | 255 | | 16.0 | | 0.26 |
| Heilongjiang | 11818 | 6.0 |  | | | 1380 | | 12.2 | | 241 | | 2.3 | | 0.02 | |
| Anhui | 55901 | 8.3 |  | | | 6658 | | 15.8 | | 2399 | | 36.4 | | 0.48 | |
| Jiangxi | 37451 | 7.6 |  | | | 4717 | | 14.7 | | 595 | | 6.6 | | 0.07 | |
| Henan | 97243 | 8.8 |  | | | 6958 | | 10.0 | | 1619 | | 20.4 | | 0.18 | |
| Hubei | 46490 | 9.7 |  | | | 3600 | | 8.3 | | 1493 | | 8.1 | | 0.07 | |
| Hunan | 85126 | 10.3 |  | | | 8902 | | 12.2 | | 1926 | | 31.9 | | 0.40 | |
| Hainan | 9184 | 7.4 |  | | | 1909 | | 25.1 | | 141 | | 33.6 | | 0.63 | |
| Inner Mongolia | 18617 | 11.0 |  | | | 838 | | 7.3 | | 14 | | 5.5 | | 0.04 | |
| Guangxi | 71687 | 9.2 |  | | | 10387 | | 14.6 | | 1552 | | 8.6 | | 0.12 | |
| Chongqing ^f^ | 24921 | 11.1 |  | | | 1417 | | 8.5 | | 800 | | - | | - | |
| Sichuan | 36452 | 6.3 |  | | | 4701 | | 16.9 | | 148 | | 13.9 | | 0.15 | |
| Guizhou | 23748 | 10.4 |  | | | 1814 | | 12.1 | | 382 | | 11.6 | | 0.15 | |
| Yunnan | 47709 | 8.6 |  | | | 2992 | | 9.2 | | 723 | | 25.2 | | 0.20 | |
| Shaanxi | 47010 | 10.3 |  | | | 4230 | | 12.9 | | 1798 | | 12.1 | | 0.16 | |
| Gansu | 41026 | 14.1 |  | | | 3018 | | 8.5 | | 39 | | 29.6 | | 0.36 | |
| Qinghai | 7411 | 17.6 |  | | | 501 | | 12.5 | | 40 | | 2.8 | | 0.06 | |
| Ningxia | 5887 | 6.3 |  | | | 648 | | 19.3 | | 690 | | 6.2 | | 0.08 | |
| Xinjiang | 30807 | 8.9 |  | | | 4537 | | 20.4 | | 14 | | 12.9 | | 0.23 | |
| Tibet ^f^ | 3 | 0.0 |  | | | 0 | | 0.00 | | - | | - | | - | |
