# Supplementary material for: Newborn hearing screening coverage and detection rates of hearing impairment across China from 2008-2016
Source: BMC Pediatr. 2020 Jul 30;20:360. doi: 10.1186/s12887-020-02257-9 (PMC7391493; doi:10.1186/s12887-020-02257-9)
Supplement: Supplementary file 3 [file 12887_2020_2257_MOESM1_ESM.docx]

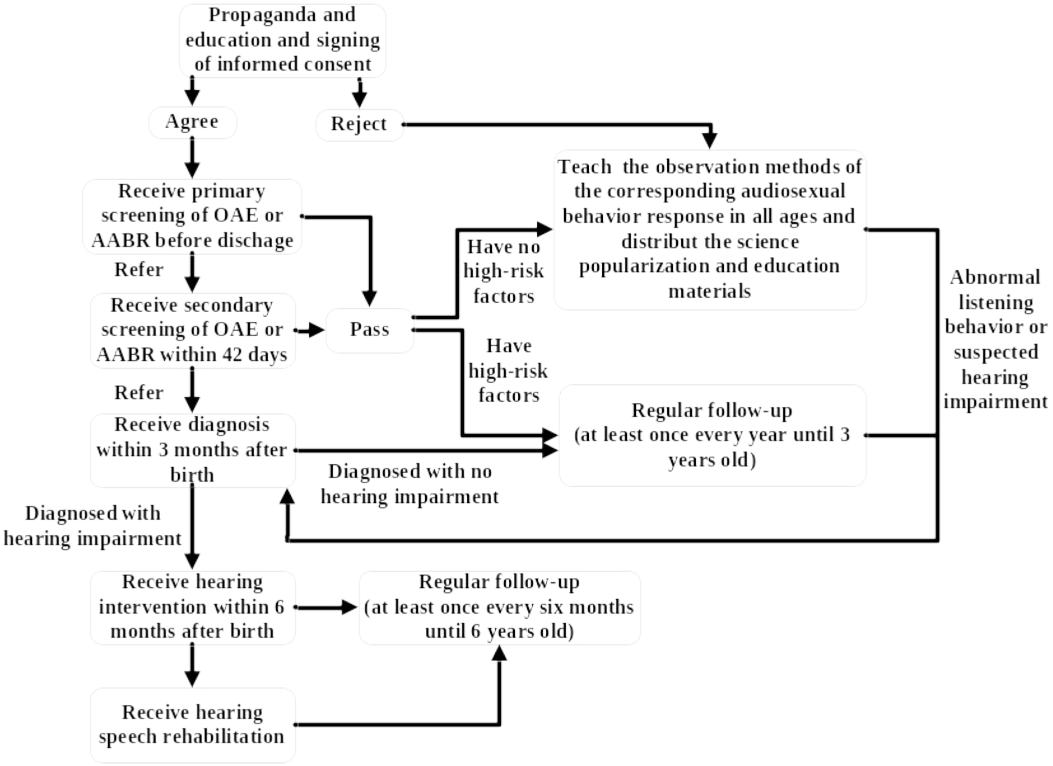
Propaganda and education and signing of informed consent
Agree
Reject
Receive primary screening of OAE or AABR before dischage
Refer
Receive secondary screening of OAE or AABR within 42 days
Pass
Have no high-risk factors
Teach the observation methods of the corresponding audiosexual behavior response in all ages and distribut the science popularization and education materials
Have high-risk factors
Abnormal listening behavior or suspected hearing impairment
Refer
Receive diagnosis within 3 months after birth
Regular follow-up (at least once every year until 3 years old)
Diagnosed with no hearing impairment
Diagnosed with hearing impairment
Receive hearing intervention within 6 months after birth
Regular follow-up (at least once every six months until 6 years old)
Receive hearing speech rehabilitation
